# Retraction: A novel bacterial isolate Stenotrophomonas maltophilia as living factory for synthesis of gold nanoparticles

**DOI:** 10.1186/1475-2859-8-52

**Published:** 2009-10-15

**Authors:** Yogesh Nangia, Nishima Wangoo, Nisha Goyal, G Shekhawat, C Raman Suri

**Affiliations:** 1 Institute of Microbial Technology (CSIR), Sector 39-A, Chandigarh 160036, India; 2 Institute of Nanotechnology, Northwestern University, Evanston, IL, USA

## Retraction

This article [1] was submitted to *Microbial Cell Factories *at the same time as a very similar article [2] was submitted to *Applied Physics Letters*, *s*ubsequently published in June 2009. The two articles were produced from the same data and contained extensive overlap including duplication of figures. As such the authors would like to retract the *Microbial Cell Factories *article. The authors would like to apologise for any inconvenience this may have caused to the editorial staff and readers.
